# Comparison of transcriptomic landscapes of different lamb muscles using RNA-Seq.

**DOI:** 10.1371/journal.pone.0200732

**Published:** 2018-07-24

**Authors:** Eileen Armstrong, Andres Iriarte, Paula Nicolini, Jorge De Los Santos, Javier Ithurralde, Alejandro Bielli, Gianni Bianchi, Francisco Peñagaricano

**Affiliations:** 1 Departamento de Genética y Mejora Animal, Facultad de Veterinaria, Universidad de la República, Montevideo, Uruguay; 2 Departamento de Desarrollo Biotecnológico, Instituto de Higiene, Facultad de Medicina, Universidad de la República, Montevideo, Uruguay; 3 Polo de Desarrollo Universitario Instituto Superior de la Carne, Centro Universitario de Tacuarembó, Universidad de la República, Tacuarembó, Uruguay; 4 Department of Animal Sciences, University of Wisconsin, Madison, Wisconsin, United States of America; 5 Departamento de Morfología y Desarrollo, Facultad de Veterinaria, Universidad de la República, Montevideo, Uruguay; 6 Independent Researcher, Paysandú, Uruguay; 7 Department of Animal Sciences, University of Florida, Gainesville, Florida, United States of America; 8 University of Florida Genetics Institute, University of Florida, Gainesville, Florida, United States of America; INIA, SPAIN

## Abstract

Transcriptome deep sequencing is a powerful tool for exploring the genetic architecture of complex traits. Gene expression patterns may explain a high degree of the observed phenotypic differences in histochemical and metabolic parameters related to meat quality among different muscles. In this study, we sequenced by RNA-Seq the whole transcriptome of nine lamb muscles: Semimembranosus (SM), Semitendinosus (ST), Cranial gluteobiceps, Gluteus medius (GM), Rectus femoris, Supraspinatus (SS), Longissimus lumborum (LL), Adductor and Psoas major. Significant gene expression differences were detected between almost all pairwise comparisons, being more pronounced between SS and ST, SM and LL, and ST and GM. These differences can be explained in terms of ATPase and glycolytic activities, muscle fiber typing and oxidative score, clustering muscles as fast glycolytic, intermediate or slow oxidative. ST showed up-regulation of gene pathways related to carbohydrate metabolism, energy generation and protein turnover as expected from a fast white muscle. SS showed myosin isoforms typical of slow muscles and high expression of genes related to calcium homeostasis and vascularization. SM, LL and GM showed in general intermediate gene expression patterns. Several novel transcripts were detected, mostly related to muscle contraction and structure, oxidative metabolism, lipid metabolism and protein phosphorylation. Expression profiles were consistent with previous histochemical and metabolic characterization of these muscles. Up-regulation of ion transport genes may account for significant differences in water holding capacity. High expression of genes related to cell adhesion, cytoskeleton organization, extracellular matrix components and protein phosphorylation may be related to meat yellowness and lower tenderness scores. Differential expression of genes related to glycolytic activity and lactic acid generation among fast, intermediate and slow muscles may explain the detected final meat pH differences. These results reveal new candidate genes associated with lamb meat quality, and give a deeper insight into the genetic architecture of these complex traits.

## Introduction

High and consistent quality are critical for the sheep meat industry, which could be achieved through improving breeding strategies and management procedures but also by identifying genes that can help to predict meat quality [1]. The latter is especially critical in sheep, as little is known about its genome and gene expression profiles compared to cattle, pig or poultry.

Meat quality involves several complex traits, difficult to improve and control. Transcriptome deep sequencing is a powerful tool for unraveling the genetic architecture of these traits. Gene expression patterns explain a high degree of the observed phenotypic variation, and thus the identification of genes and metabolic pathways involved could help to improve this product [2–4].

Previous studies of gene expression profiles in sheep muscle include comparisons between different breeds through RNA-Seq [5–7], and muscle microarray-based studies of callipyge vs. wild-type lambs [8, 9] and microRNAs [10]. Sun et al. (2016) found 960 genes differentially expressed (DE) in Longissimus dorsi muscle from two Chinese sheep breeds that differ greatly in meat production traits, mostly related to muscle growth, muscle cell differentiation, metabolic properties and cellular components [7]. In a similar study comparing muscle Biceps brachii in divergent sheep breeds, most DE genes were involved in structural and metabolic pathways [5, 6]. Differences in gene expression between callipyge and wild-type lambs related mostly to muscle fiber type conversion and muscle growth [8, 9]. Zhang et al. (2015) found 22 miRNA highly expressed in sheep Biceps femoris muscle, some of which were associated with meat production performance [10]. To our knowledge, there are no studies that compare general expression profiles of different sheep muscles under normal conditions, as has been done in other species [11].

In mouse, transcriptome deep sequencing of a fast muscle (Quadriceps) in comparison with a slow one (Soleus) showed pronounced differences in overall gene expression, especially in genes related to muscle contraction, carbohydrate metabolism and different types of non-coding RNA [11].

Mammalian skeletal muscles differ in their contractile and metabolic properties, mainly related to speed of contraction and resistance to fatigue. In a previous study, Ithurralde et al. (2015, 2017) studied fiber content and type among lamb muscles with different histochemical and meat quality characteristics [12, 13]. The authors demonstrated the existence of specific intermuscular differences in fiber typing and meat quality in sheep. How these properties relate to gene expression profiles and ultimately to meat quality is largely unknown, and this knowledge is of great interest for the meat industry. In this study we sequenced the whole transcriptome of nine different muscles that belong to highly valuable cuts in unrelated male lambs using RNA-Seq technology, in order to associate gene expression patterns with histochemical properties, metabolic parameters and meat quality characteristics. Our results indicate that transcription profiles differ between muscles, especially according to their metabolism and main fiber types, and that the detected gene expression differences may explain part of the muscle phenotypic variation observed.

## Materials and methods

### Sample collection, muscle fiber typing and meat quality measures

All animal experimental procedures were approved by the Animal Experimentation Committee (CHEA, Universidad de la República, Montevideo, Uruguay). Muscle samples were collected in a previous study by Ithurralde et al. (2015, 2017) [12, 13] from four unrelated Poll Dorset cross-bred lambs, raised on grasslands. Animals were acquired from different flocks. After weaning they received soy crop and grain sorgum supplementation until slaughter, with final live weights ranging from 67 to 72 kg at 14 months old. Samples were collected immediately (within 30 minutes) after slaughter from the mid superficial belly of the following muscles: Semimembranosus (SM), Semitendinosus (ST), Cranial Gluteobiceps (GB), Gluteus medius (GM), Rectus femoris (RF), Supraspinatus (SS), Longissimus lumborum (LL), Adductor (AD) and Psoas major (PM). The anatomical location of the muscles sampled is presented as supplementary material (S1 Fig). Samples were embedded in RNAlater solution (Ambion) and stored at -20°C until RNA extraction.

Histochemical tests for fiber typing, fiber morphometric analysis and meat quality determination were performed by Ithurralde et al. (2015, 2017)[12,13].

### RNA extraction, library generation and sequencing

Muscle samples were frozen in liquid nitrogen and thoroughly ground to powder using mortar and pestle. Total cellular RNA was isolated and purified with TRIzol® Reagent in conjunction with PureLink RNA Mini Kit (Cat.12183018A). To avoid genomic DNA contamination, eluted RNA was treated with DNase-free DNase Set (QIAGEN). RNA yield and quality were measured through an Agilent 2100 Bioanalyzer (Model G2939B).

Libraries from individual samples were sequenced with Illumina’s HiSeq 2000 at Macrogen (Korea), by means of 100 pb paired-end reads, in 4 lanes and 9 samples per lane. Libraries were barcoded and multiplexed before sequencing.

### Mapping reads to the sheep reference genome

Sequencing reads were mapped to the sheep (*Ovis aries*) reference genome (Oar_v3.1) using the software Tophat (v2.0.13) [14, 15]. Following [16] and [17], two consecutive rounds of alignments were performed in order to maximize sensitivity to splice junction discovery. Briefly, novel splice junctions were initially discovered in each sample, and then these novel splice junctions plus known splice junctions from Ensembl annotation were combined and supplied to Tophat for a second round of alignment. This strategy allows a full utilization of the novel junctions identified in the samples.

### Assembly of the transcripts and estimation of the abundance

Transcript models were reconstructed using Cufflinks (v2.2.1) [18]. This software recreates a set of transcript models that best explain the sequencing alignments observed in the samples. The reference ovine annotation (GTF file) was merged with sample assemblies with the purpose of combining annotated (known) transcripts with novel transcript units. This approach maximizes the quality of the final assembly [16, 17]. Finally, in each muscle sample, the total number of reads that effectively mapped to each gene described in the final assembly was calculated using the python script *htseq-count* [19].

### Gene expression analysis

Differentially expressed genes (DEG) between muscles were detected using the *R* package *edgeR* (v.3.12.0) [20]. Briefly, *edgeR* uses the trimmed mean of M-values (TMM) as RNA-Seq normalization method, calculates gene wise negative binomial dispersion parameters using an empirical Bayes procedure, and fits generalized linear models followed by likelihood ratio tests for detecting differentially expressed genes [21]. This study consisted in a paired design because pairwise comparisons were performed between muscles, and each animal in the study posed both muscles. In this context, the likelihood ratio test detected genes that were differentially expressed between muscles, adjusting simultaneously for differences between animals. This test can be viewed as a generalization of a paired t-test. This experimental design increases the statistical power and reduces potential confounder effects. The *P*-values were adjusted for multiple testing using the Benjamini-Hochberg procedure [22].

Gene and protein functions were taken mainly from Ensembl browser and UniProt database (http://www.uniprot.org/).

### Gene set enrichment analysis

The significant enrichment of Gene Ontology (GO) functional terms with genes that showed differential expression between muscles of interest was analyzed using a Fisher’s exact test. Significant genes with FDR ≤ 0.05 and Ensembl annotations were tested against the background set of all expressed genes with Ensembl annotations. The analysis was performed using the *R* package *goseq* [23].

### Validation by RT-PCR

Eight genes that showed highly significant differential expression between ST and SS (the most contrasting muscles in terms of metabolic and histochemical properties according to Ithurralde et al., 2015, 2017 [12, 13]) were chosen for validation of the RNA-Seq results: myozenin 2 (*MYOZ2*), myosin light chain 2 (*MYL2*), myosin light chain 3 (*MYL3*), tropomyosin 3 (*TPM3*), myosin light chain 6 (*MYL6*), troponin T1 (*TNNT1*), troponin C1 (*TNNC1*) and methyl transferase-like 21C (*METTL21C*). The same muscle samples used for RNA-Seq were used for validation of differential expression. The validation was performed using quantitative real-time PCR (qPCR) conducted with theCorbett Rotor-Gene 6000 system (QIAGEN). A total of 1μg RNA from each sample was used to synthesize cDNA using the SuperScript®III Transcriptase (Invitrogen) with primers Oligo-dT (Invitrogen) following the manufacturer’s instructions. Primers (S1 Table) to specifically amplify cDNA of target genes were designed using the Primer3 software (http://bioinfo.ut.ee/primer3/) based on ovine genome sequences (Oar_v3.1/oviAri3) available from UCSC Genome Browser (https://genome.ucsc.edu). Primers were designed to cross exon-exon junctions to minimize the potential of amplifying genomic DNA. Real-time PCR reactions were performed in a total volume of 15 μl containing 2 uL (20 ng) cDNA, 1uL forward and reverse primer mix (300nM each), 4.5 uL PCR water and 7.5 μL QuantiFast SYBR Green 2x qPCR Master Mix, QIAGEN), using the following standard amplification conditions for all sets of primers: 5 min at 95°C and 40 cycles of 15 s at 95°C, 45 s at 60°C and 20 s at 72°C. Dissociation curves were run on all samples to detect primer-dimers, contamination or presence of other amplicons. For each gene assessed, samples were run in triplicate. Each run disc included a triplicate pool of cDNA from all ST and SS samples assayed (exogenous control) and a duplicate non-template control (NTC). Gene ACTB (β-actin) [24] was used as the housekeeping gene (endogenous control). Gene expression was measured by relative quantification (2-ΔΔCt method, [25]) to the exogenous control and normalized to the endogenous control. Amplification efficiencies (E = [10-1/slope-1]) for all genes were estimated by linear regression (Rotor Gene 6000 Q Software 1.7.75) of a triplicate cDNA standard curve (n = 5 dilutions, from 100 to 6.25ng/tube). Amplification efficiencies ranged from 0.94 to 1.10. The intra-assay %CVs between triplicates were < 2%.

## Results and discussion

### Overall evaluation of gene expression in different muscles

Whole transcriptomes from nine different muscles of four unrelated cross-bred lambs were successfully sequenced using RNA-Seq technology. Sequencing data can be accessed through NCBI GEO with accession number GSE112500. A summary of sequencing read alignments to the reference genome is presented in S2 Table. The number of uniquely mapped reads that were effectively used for the gene expression analysis ranged between 4 and 6 million per sample. This number of mapped reads is enough to accurately quantify moderate to highly expressed genes (e.g., [26]). Indeed, for gene expression, sequencing depth is much less important than replication [27], and so, we preferred to include more animals (i.e. more biological replicates per muscle) than increasing read coverage.

**Fig 1:**
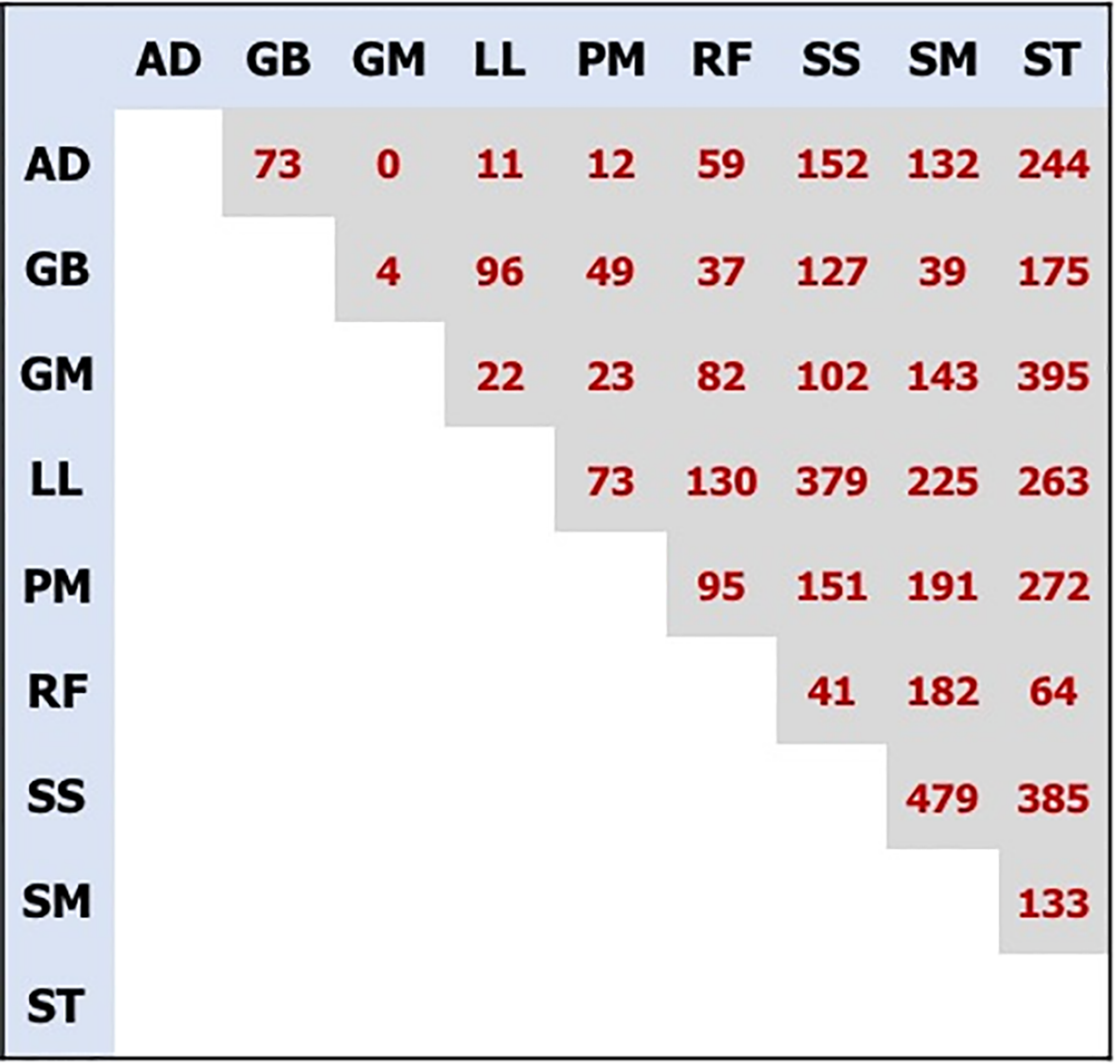
Total number of significant genes detected in each pairwise comparison. Controlling false discovery rate (FDR) at 1%, the matrix shows the total number of genes that showed at least a 2-fold expression difference (Fold Change > 2) in each pairwise comparison. SM: Semimembranosus, ST: Semitendinosus, GB: cranial Gluteobiceps, GM: Gluteus medius, RF: Rectus femoris, SS: Supraspinatus, LL: Longissimus lumborum, AD: Adductor, PM: Psoas major. (File: Fig 1.tiff).

Controlling false discovery rate (FDR) at 1%, Fig 1 shows the total number of genes that showed at least a 2-fold expression difference (Fold Change > 2) in each pairwise comparison. Significant differences were detected between almost all muscle comparisons. While some muscles show very similar expression patterns, with no genes differentially expressed (AD *vs*. GM) or very few (e.g. 4 genes between GB and GM, 11 genes between AD and LL, and 12 between AD and PM), some others show striking differences; for example, there were 479 differentially expressed genes between SM and SS, 395 between ST and GM, 385 between SS and ST, and a total of 379 between SS and LL.

**Fig 2:**
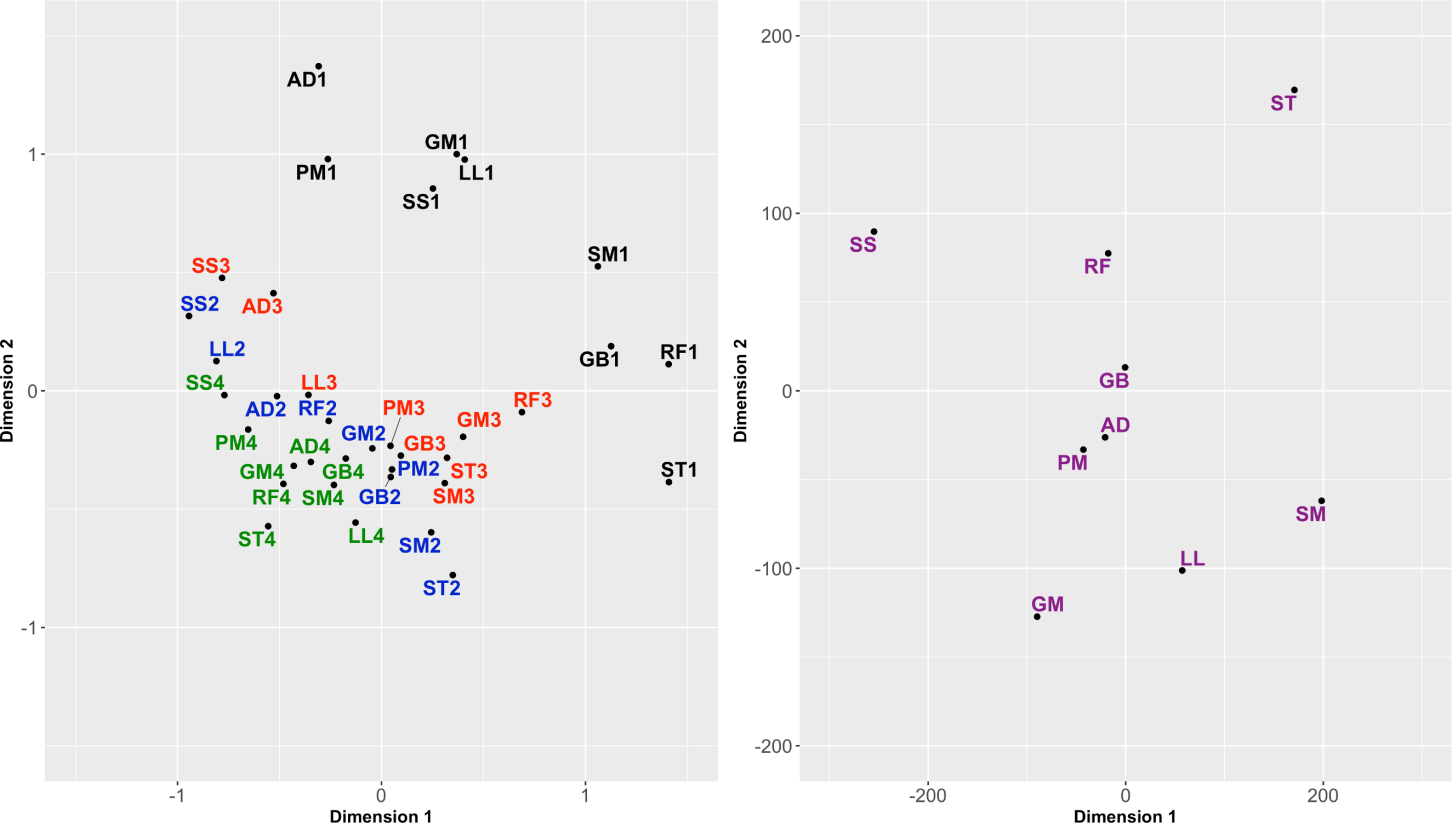
Multidimensional scaling (MDS) plot showing the relative similarities of the muscles under study. Using all the samples (left) and using the set of differentially expressed genes (right). SM: Semimembranosus, ST: Semitendinosus, GB: cranial Gluteobiceps, GM: Gluteus medius, RF: Rectus femoris, SS: Supraspinatus, LL: Longissimus lumborum, AD: Adductor, PM: Psoas major. (File: Fig 2.tiff).

Alternative multidimensional scaling (MDS) analyses were performed in order to visualize the overall relationship between the samples under study (Fig 2). The MDS plot displaying the 36 samples clearly shows that the greatest variation is explained by the animal effect, but within each animal, the muscles are usually separated following the same pattern, e.g. SS and ST are consistently very different (Fig 2, left). Note that this is a paired design study, and hence, differentially expressed genes were detected adjusting simultaneously for differences between animals. The MDS plot based on the set of DEG shows that both dimensions clearly separate muscles SS, SM, ST, GM and LL, with the other four muscles in intermediate positions (Fig 2, right).

There is a great agreement between dimension 1 of the MDS plot and the ATPase (contraction speed) and glycolytic activities of these muscles; in particular, SS has the lowest ATPase activities and also the lowest glycogen synthetase, phosphorylase, and phosphofructokinase activities, while SM and ST have the highest ATPase and also the highest phosphorylase, glycogen synthetase, and phophofructokinase activities [28]. Dimension 2 clearly separates SM, LL and GM from ST and SS. According to [28], SM and Longissimus dorsi are considered fast twitch red muscles, having characteristics typical of fast muscles (high ATPase and high glycolytic activity) but also of slow muscles (high oxidative activity). Ithurralde et al. (2015) found LL (lumbar portion of Longissimus dorsi) within the cluster of fast glycolytic muscles, as well as GM, and clustered SM within fast red muscles [12]. However, the three muscles showed high oxidative fiber proportion and high oxidative scores, as well as high glycolytic fiber proportion. Although being a fast-glycolytic muscle, ST showed extreme values and significant differences in terms of fiber type and morphology in comparison with both GM and LL [12].

Interestingly, MDS plots showed a very similar pattern to the principal component analysis presented by Ithurralde et al. (2017), in which they study the general relationships between meat quality traits and fiber typing across muscles [13]. In agreement with the present gene expression analysis, the muscles clustered in three main categories, with SS, ST and SM clearly separated. SS showed typical values of slow oxidative muscles, SM is an intermediate fast red muscle, and ST is a typical fast glycolytic muscle [12]. As will be seen later on, the observed differences in gene expression profiles could well be related to these metabolic and histochemical properties.

### Functional characterization of differently expressed genes between pairs of muscles

The following analyses will focus on the muscles that showed the highest differences in terms of gene expression (ST, SS, SM, LL and GM).

Table 1 shows the 30 most significant differentially expressed genes (threshold: FDR < 1%) between ST and SS, all of them with fold change > 2 (log_2_FC >1). Several genes that were up regulated in SS are related to muscle tissue structure, including myosin heavy chain 7B and troponin T1, C1 and I1, all common isoforms of slow muscle cells [29], and in agreement with previous transcriptome studies in sheep muscle [6] and in mice [11].

**Table 1:** Top 30 most significant genes detected in Semitendinosus (ST) *vs*. Supraspinatus (SS) pairwise comparison. Positive log_2_ fold change (Log_2_FC) means higher expression in ST *vs*. SS.

Functional Characterization	Gene Name	Chr.	Coordinates	Log_2_FC	P value
Myofibril assembly; actin binding.	MYOZ2	6	6265305–6302669	-4.29	2.95E-41
Regulation of transcription, development and morphogenesis.	HOXD8	2	132876251–132877353	4.17	3.96E-31
Muscle fiber development; myofibril assembly; striated muscle contraction.	MYL2	17	54295568–54303068	-3.78	6.04E-30
Regulation of striated muscle contraction; muscle structure development.	MYL3	19	52560400–52569198	-4.25	8.08E-29
Sarcomere organization.	MYLK3	14	4550957–14594827	-3.74	1.73E-27
Muscle contraction; cytoskeleton organization.	TPM3	1	103049267–103067657	-3.20	9.56E-27
Muscle fiber organization.	MYL6	3	163096468–163101729	-3.84	2.44E-25
Carbohydrate catabolic process; protein phosphorylation.	CHI3L1	12	352663–366834	3.77	7.03E-25
Development and morphogenesis; neuromuscular processes.	HOXC10	3	132378989–132383129	7.33	3.36E-22
Cell differentiation; cytoskeleton organization.	CCDC63	17	54257918–54295514	-3.80	3.97E-21
Cytoskeleton protein; cell-cell adhesion.	PDLIM1	2	239847424–239852355	-1.75	8.12E-20
Regulation of skeletal muscle contraction; transition fast to slow fibers.	TNNT1	14	59548305–59556856	-3.24	3.37E-19
Motor activity.	MYH7B	13	63837807–63862044	-4.29	4.05E-19
Sarcoplasmic reticulum calcium ion transport; transition fast to slow fibers; muscle cell development; regulation of muscle contraction.	ATP2A2	17	53803322–53859050	-2.82	7.37E-19
Cell to cell adhesion.	TMEM2	2	64376666–64436803	1.44	1.26E-18
Post transcriptional modifications.	U6 snRNA	14	25336001–25350065	-4.35	2.31E-18
Regulation of skeletal muscle contraction; transition fast to slow fibers; regulation of muscle filament sliding speed.	TNNC1	19	48403558–48406316	-3.21	3.92E-18
Transition fast to slow fibers; striated muscle contraction and development.	TNNI1	12	78591681–78596742	-3.40	7.18E-18
Regulation of release of sequestered calcium ion into cytosol by sarcoplasmic reticulum; regulation of membrane repolarization; sarcomere organization.	CASQ2	1	92171986–92243108	-2.74	6.28E-16
	NTU	1	230473614–230478546	-4.39	3.33E-15
Protein methylation.	METTL21C	10	78274732–78283255	-5.25	6.10E-15
Sarcomere organization.	IGFN1	NA	NA	3.26	7.48E-15
Regulation of skeletal muscle contraction; transition fast to slow fibers; regulation of slow-twitch skeletal muscle fiber contraction and filament sliding.	OMYHCS	NA	NA	-3.50	1.11E-14
Chemical synaptic transmission; negative regulation of phosphatase activity.	DLG2	21	10779194–11743008	-2.65	2.29E-14
Sarcomere organization.	MYOM3	2	241691327–241739483	-2.98	2.40E-14
Ephitelium morphogenesis; cell communication; extracellular matrix.	FREM2	10	23560959–23715430	-2.57	3.18E-14
Glucose homeostasis.	STXBP5L	1	183898068–184274141	2.78	6.88E-14
Skeletal system development.	KIAA1217	13	24120977–24476577	-1.54	1.20E-13
Regulation of oxidative metabolism.	NTU *(ESSRG)*	12	18687137–18712068	-2.40	1.93E-13
Fatty acids transport.	FABP3	2	235135457–235145925	-2.35	2.83E-13

NTU: Novel Transcript Unit. Chr.: chromosome. NA: not available at OAR v.3.1.

*ATP2A2* and *CASQ2* genes are related to calcium ion homeostasis. ATP2A2 enzyme is a slow muscle isoform that transports calcium between the cytosol and the sarcoplasmic reticulum lumen, and is involved in the regulation of contraction and relaxation [30]. CASQ2 is the slow skeletal muscle isoform of calsequestrin, a calcium-binding protein that works as a calcium store in muscle cells, regulating the release of calcium through ryanodine channels [31]. Even if calcium channels are more densely distributed in fast fibers than in slow ones (to accelerate charge movement), calcium entry through the sarcolemma is higher in slow fibers, at rest and during depolarization [29]. Our findings support the importance of calcium homeostasis in a slow twitch muscle as SS, and later on its implications on water holding capacity will be discussed.

Troponins, myosin and *ATP2A2* are also involved in the transition between fast and slow fibers [29]. This could be related to muscle plasticity, a property of the muscle fibers that can change their characteristics in response to external influences, specially to nerve activity [29]. Myosin gene expression is also related to fiber type changes and abundance [8]. Up-regulation of genes related to myofiber type and composition in SS shows a higher fiber heterogeneity of slow muscles with respect to fast muscles, as was observed in RNA-Seq studies in mice [11].

On the other hand, genes more expressed in ST are related to morphogenesis (*HOXD8* and *HOXC10*, [32], carbohydrate catabolic processes and protein phosphorylation (*CHI3L1*, [33], and glucose homeostasis (*STXBP5L*, [34], among others, all functions associated with high metabolic activity and tissue development in a typical fast-twitch muscle. High expression of genes related to morphogenesis and development were also found in the transcriptome of Chinese lamb muscles [6], and may be related to growth in young animals.

Accordingly, Ithurralde et al. (2015) found, in the histochemical analyses performed in the same muscle samples used for the present study, a typical slow muscle fiber typing profile for SS (the highest percentage of type I fibers and the lowest percentage of type II and glycolytic fibers, combined with a high oxidative score) and a typical fast glycolytic profile for ST (the lowest percentage of type I fibers and of oxidative fibers, but the highest percentage of type II and glycolytic ones) [12].

Two Novel Transcript Units (NTU, unknown/unannotated ovine genes) were detected in ST *vs*. SS comparison (Table 1). The first NTU of the table (log_2_FC = -4.38, P value = 3.33e-15) mapped to OAR1 (chr1: 230473614–230478546). This is a conserved region according to Ensembl browser comparative genomics chart, flanked by *PLCH1* and *MME* genes, as well as by two ncRNA. A CDS (Ensembl annotation RP11-451G4.2) and a long intervening no coding RNA (lincRNA; RP11-451G4.3) were reported for this syntenic region in the human genome. According to GO annotations in Ensembl browser and UniProt database, there is RNASeq evidence that the human CDS is differentially expressed in muscle, related to slow-twitch skeletal muscle fiber contraction regulation and calcium ion transport, two functions associated with its up regulation in SS. The other NTU (*ESRRG*) mapped to OAR 12 (chr12: 18687137–18712068), in a conserved region flanked by *ESRRG* and *GPATCH2* genes. In the human homologous region there is an intron shared by two *ESRRG* isoforms (Estrogen-Related Receptor γ). This is a remarkable gene as it is considered a key regulator of oxidative metabolism in skeletal muscle, increasing mitochondrial activity. It is up regulated in slow-twitch type I fibers, required for long term adaptation to exercise, and induces expression of several other genes related to calcium ion transport, muscle contraction, fatty acid metabolism and angiogenesis [35]. In the present study we found several genes up regulated in SS directly related to these functions, as can be seen in Table 1.

**Fig 3:**
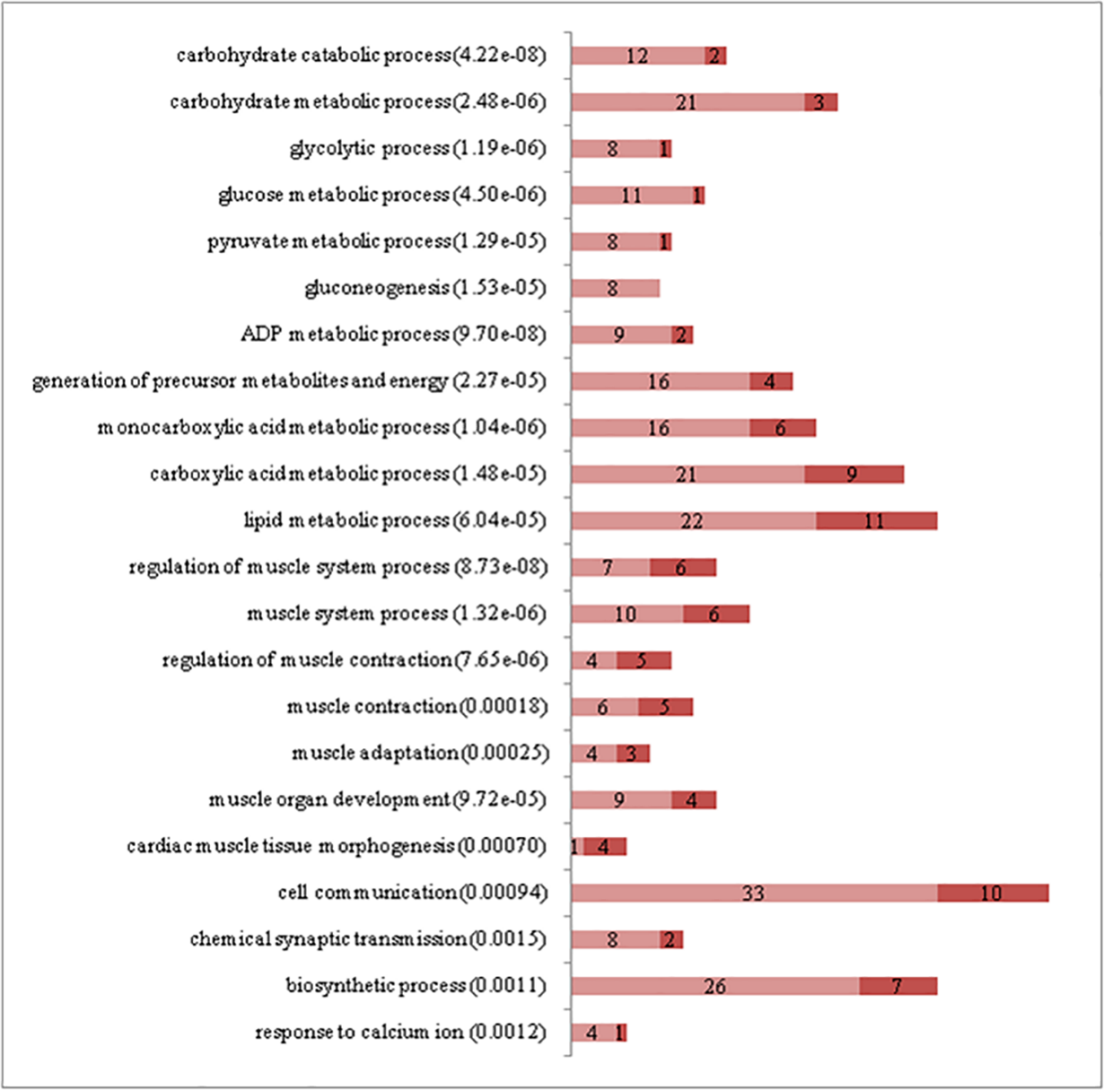
Gene Ontology biological process terms significantly enriched with differentially expressed genes between Semitendinosus (ST) and Supraspinatus (SS). The graphs show the number of differentially expressed genes in each functional category. P values are in parentheses. Genes up-regulated in ST are in light red while genes up-regulated in SS are in dark red. (File: Fig 3.tif).

The significant enrichment of Gene Ontology (GO) terms with differentially expressed genes was evaluated using a Fisher´s exact test. Fig 3 displays the most relevant functional categories that were over-represented with significant genes detected in ST *vs*. SS pairwise comparison. Important differences were observed in functional terms closely related to energy generation and carbohydrate metabolic processes; as expected, all these terms were up-regulated in ST compared to SS, in accordance with fast-glycolytic metabolism. ST also showed higher expression of genes related to carboxylic acids and lipid metabolism, which may include amino acids and fatty acids related to protein turnover and energy expenditure. As seen from the most DE genes analysis (Table 1), processes related to muscle function and its regulation were also active in both muscles. ST showed up regulation of genes related to cell communication, which includes cell-cell adhesion and attachment to the extracellular matrix, and to biosynthetic processes. Although there were more genes related to calcium transportation within the top 30 DEG in SS than in ST (Table 1), the latter showed more genes associated with response to calcium ions, consistent with the fact that fast glycolytic muscles need to ensure quick charge movements to allow high speed of contraction [29].

Table 2 shows the most differently expressed genes between SM (fast red muscle) and SS (slow red muscle). Some of the genes that were up regulated in SM are related to morphogenesis and development (*HOXD8* and *HOXC10* [32]), carbohydrate catabolic processes (*CHI3L1*, [33]), protein phosphorylation (*CHI3L1* and *KNDC1* [33, 36]), lactate transport (*SLC16A3*, [37]), glycogen metabolism (*PPP1R3D*, [38]) and muscle contraction (*SNTB1*, [39]); all of them typical features of fast muscles. On the other hand, SS showed higher expression of two genes related to calcium transport (*ATP2A2* [30] and *PVALB* [40]), similar to what was observed in the comparison ST *vs*. SS, one to sodium transport (*ADRB2* [41]), and a gene that encodes a potassium channel, *KCNMA1* [42]. Potassium is less important than other ions for muscle contraction, but slow muscle fibers are more active than fast ones in removing potassium from the extracellular space [29]. Both SM and SS showed up regulation of genes related to muscle contraction and cell proliferation. This may be associated with the intermediate characteristics of SM compared to SS and ST, combining contractile and metabolic properties of both fast and slow muscles. In the former fiber typing study by Ithurralde et al. (2015), SM had similar values than ST for type I and type II fibers, but is more similar to SS in terms of oxidative and glycolytic fibers percentage and oxidative score [12].

**Table 2:** Top 30 most significant genes detected in Semimembranosus (SM) *vs*. Supraspinatus (SS) pairwise comparison. Positive log_2_ fold change (Log_2_FC) means higher expression in SM *vs*. SS.

Functional Characterization	Gene Name	Chr.	Coordinates	Log_2_FC	P value
Carbohydrate catabolic process; protein phosphorylation.	CHI3L1	12	352663- 366834	3.96	5.69E-27
Regulation of transcription; development and morphogenesis.	HOXD8	2	132876251–132877353	3.85	1.23E-25
Development and morphogenesis; neuromuscular processes.	HOXC10	3	132378989–132383129	7.25	2.99E-21
Adrenergic receptor signaling pathway; regulation of sodium channels; thermogenesis.	ADRB2	5	57750711–57751970	-1.41	6.11E-17
Muscle contraction.	NTU *(MYBPC1)*	3	170476766–170478580	-1.77	1.75E-15
Protein phosphorylation.	MYLK4	NA	NA	-3.36	1.58E-13
Striated muscle tissue development; protein dephosphorylation and sumoylation.	EYA1	9	47588481–47960610	-2.57	3.45E-13
Regulation of transcription; negative regulation of cell proliferation.	SMYD2	12	67363929–67396799	1.27	2.52E-12
Muscle contraction.	SNTB1	NA	NA	1.61	2.66E-12
Protein methylation.	METTL21C	10	78274732–78283255	-4.39	5.23E-12
Cellular potassium ion homeostasis.	KCNMA1	25	32480589–32906604	-1.07	9.55E-12
Adrenergic receptor signaling pathway; regulation of sodium channels; thermogenesis.	NTU *(ADRB2)*	5	57760913–57768772	-1.78	1.71E-11
Protein phosphorylation, ATP binding.	NTU *(MYLK4)*	20	49762606–49765006	-4.46	4.20E-11
Microtubule binding; intracellular transport.	CLIP4	3	35840677–35897004	-1.23	8.03E-11
	NTU	3	138839190–138842956	4.17	8.40E-11
Regulation of protein phosphorylation and GTPase activity.	KNDC1	22	50434284–50485217	1.07	8.53E-11
Lipid metabolism, angiogenesis and muscle cell differentiation.	NTU *(RORA)*	7	46246545–46258218	-1.35	8.80E-11
Regulation of cytosolic calcium ion concentration.	PVALB	3	180019349–180067626	-4.97	1.64E-10
	NTU	2	220339844–220346024	-1.60	2.17E-10
	NTU	NA	scaffoldJH922385.1:3193–4805	-3.33	2.96E-10
Transcription regulation; cell diferentiation; development.	SIM2	1	267018135–267070595	2.36	4.06E-10
Regulation of synaptic membrane exocytosis; cAMP-mediated signaling; insulin secretion.	RIMS2	9	72976729–73464816	2.82	4.54E-10
Protein phosphorylation; cytoskeleton organization.	MAST2	1	20357495–20388272	-1.37	5.49E-10
Lipid metabolism, angiogenesis and muscle cell differentiation.	NTU *(RORA)*	7	46275748–46290277	-1.19	6.80E-10
Lactate transmembrane transport.	SLC16A3	11	49799205–49803627	1.66	6.83E-10
Cell proliferation and regulation of gene expression.	ZFP36L1	7	77522194–77525646	0.96	9.51E-10
Microtubule cytoskeleton organization and cell proliferation.	TACC2	22	40850131–41052894	-1.26	1.43E-09
Lipid metabolism, angiogenesis and muscle cell differentiation.	NTU *(RORA)*	7	46258368–46267107	-1.31	1.49E-09
Sarcoplasmic reticulum calcium ion transport; transition between fast and slow fiber; T-tubule organization; muscle cell development; regulation of muscle contraction.	ATP2A2	17	53803322–53859050	-1.85	1.55E-09
Glycogen metabolic process.	PPP1R3D	13	55922753–55923652	1.64	1.58E-09

NTU: Novel Transcript Unit. Chr.: chromosome. NA: not available at OAR v.3.1.

There were nine NTUs detected in this pairwise comparison, all except one up regulated in SS (Table 2). Six could be assigned to a particular gene (*MYBPC1*, slow muscle type myosin binding protein C; *ADRB2*, beta-2 adrenergic receptor; *MYLK4*, myosin light chain kinase 4; and *RORA*, RAR related orphan receptor A). The fourth NTU (the only one that is up-regulated in SM) mapped to an intergenic region near a gene that encodes a collagen chain (*COL2A1*). The sixth NTU (Log_2_FC = -1.60; P value = 2.17e-10) mapped to OAR 2 (chr2: 220339844–220346024) in a region flanked by *DES* gene (desmin, a muscle-specific protein of the sarcomere [43]) and a non-characterized gene (ENSOARG00000020189) that in cattle and human is annotated as *SPEG* gene, related to protein phosphorylation, muscle cell differentiation and development [44]. The seventh NTU mapped to an uncharacterized region with no annotations nor chromosome assignment in OAR v.3.1. Three NTUs mapped to very close regions on OAR 7 that in the human genome fall within *RORA* gene. This gene encodes a transcription factor that plays an important role in lipid metabolism, angiogenesis and muscle cell differentiation, especially in red muscles [45].

It is interesting to note that several genes related to the transportation of calcium and other ions were up regulated in SS with respect to SM. This could be related to the significant differences in water holding capacity that were found between these muscles by Ithurralde et al. (2017)[13]. Cooking loss was 34.6% in SS and 27.6% in SM (p value < 0.05) while expressed juice (water expelled after compression) was 15.9% and 10.7%, respectively (p value < 0.05). Accelerated calcium release into the sarcoplasm increases the rate of pH decline, favors protein denaturation and induces low water holding capacity, producing meat of lower quality [46]. Although Ithurralde et al. (2017) found lower final pH values in SM than in SS (p < 0.05), this process is more affected by the rate of decline than by the final value itself [13].

Similar results can be seen for ST *vs*. SS comparison, were again there were more calcium transport related genes up regulated in SS. One of these genes is *CASQ2*, a calcium release regulator through ryanodine channels that is associated with PSE (pale, soft and exudative) meat in pigs [31, 46]. Significant differences between SS and ST were found for expressed juice, but not for cooking loss [13]. These genes could be also associated to sarcomere length, as calcium transport to and from the sarcoplasmic reticulum in *post-mortem* muscle is one of the proposed mechanisms to explain observed differences in muscle susceptibility to cold-shortening [47]. In accordance with this, Ithurralde et al. (2017) found significant differences in sarcomere length between SS and ST. These gene expression patterns could also affect calpains, calcium-dependent proteases responsible for meat tenderization, but no significant differences in Warner-Bratzler shear force (WBSF) were found between SS, SM and ST (p > 0.05; [13]).

**Fig 4:**
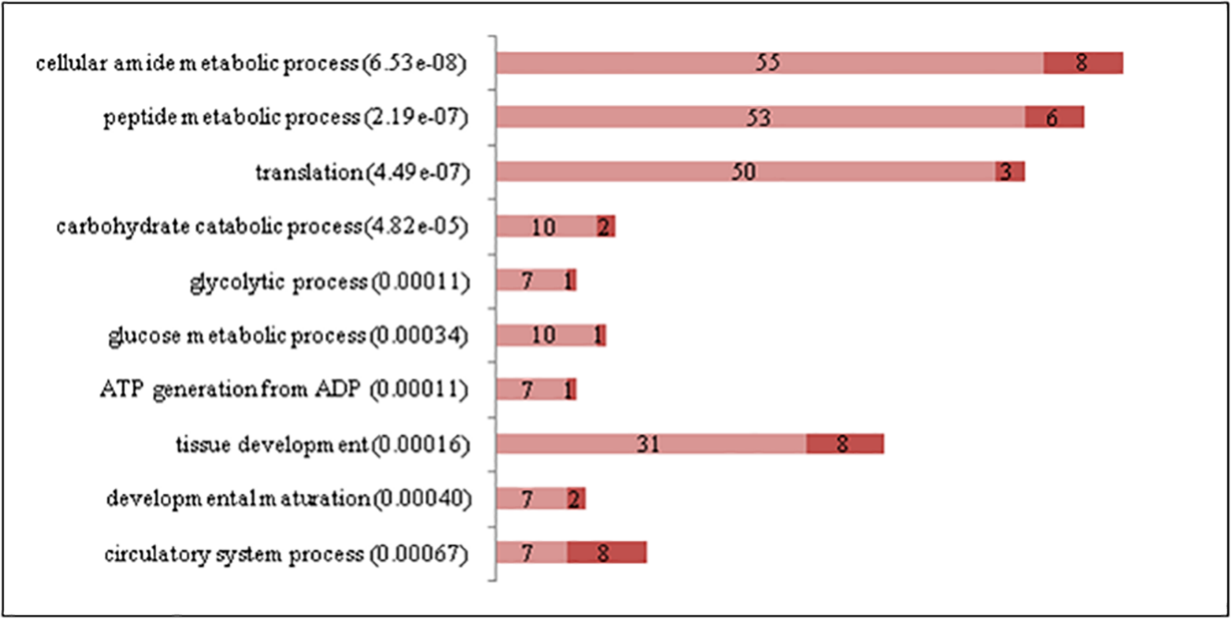
Gene Ontology biological process terms significantly enriched with differentially expressed genes between Semimembranosus (SM) and Supraspinatus (SS). The graphs show the number of differentially expressed genes in each functional category. P values are in parentheses. Genes up-regulated in SM are in light red while genes up-regulated in SS are in dark red (File: Fig 4.tif).

Few GO terms were significantly enriched with DEG from the SM *vs*. SS comparison (Fig 4), in accordance with some similar metabolic and histochemical properties of these muscles [12]. SM showed a very strong up regulation of amide and peptide metabolic processes, especially related to translation and peptide synthesis. These differences could be related to a higher protein turnover and metabolic rate in a fast-red muscle in comparison to a slow-red one. There were also significant genes associated with the circulatory system, probably related to the high vascularization of red muscles [12].

The most significant differentially expressed genes between LL and SS are shown in S3 Table. LL showed some similarities with ST, with respect to the high expression of *HOXD8*, *HOXC10* and *CHI3L1*, and also to SM with up-regulation of *SIM1*, *SNTB1* and *SMYD2*, all development-related genes [48, 39, 49]. Being the lumbar portion of Longissimus dorsi muscle, LL can either be considered as a fast-glycolytic muscle [12] or a fast-oxidative muscle [28], which may explain our findings. LL showed higher expression of a potassium channel subunit (*KCNS3*) that combined with *KCNB1*, another channel subunit more abundant in slow and fast red fibers, generates an important Na-K pump during repetitive electrical activity [29]. SS showed higher expression of genes related to muscle tissue structure and development, including *MYL2*, *MYL6*, *MYOZ*, *PDLIM1* and *EYA1* [29, 50]. Two genes related to synaptic transmission were found differentially expressed in SS *vs*. LL: genes *PLCL2* (higher expression in LL) and *DLG2* (higher expression in SS) [51, 52]. These genes could be involved in the neuromuscular junction, where motoneurons and muscle fibers interact to make possible muscle contraction and movement, both in slow and fast muscles [29].

**Fig 5:**
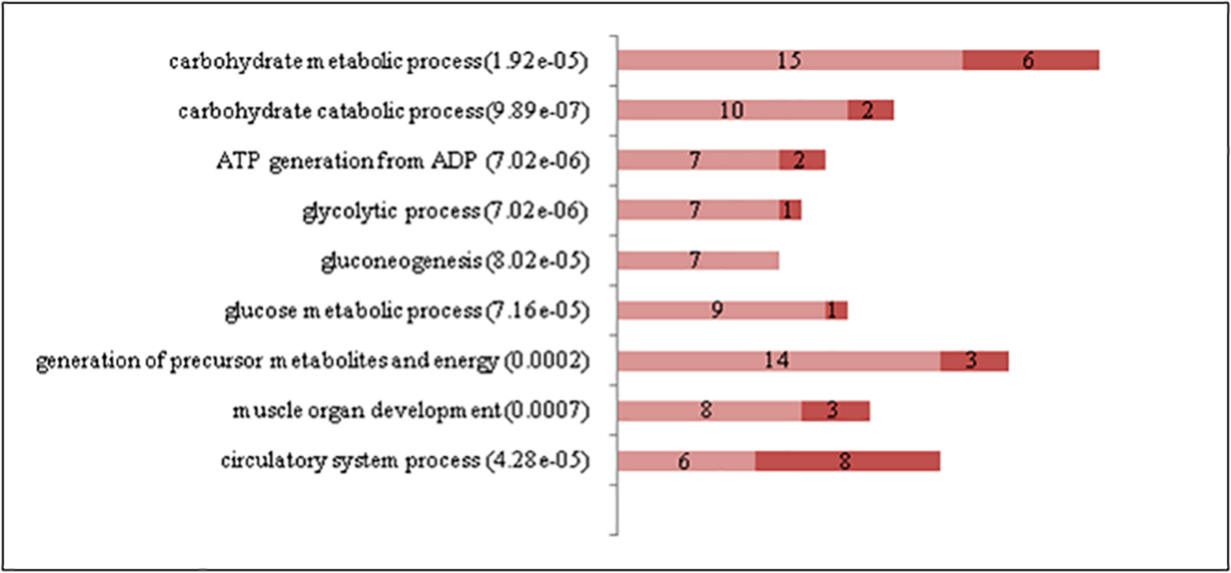
Gene Ontology biological process terms significantly enriched with differentially expressed genes between Longissimus lumborum (LL) and Supraspinatus (SS). The graphs show the number of differentially expressed genes in each functional category. P values are in parentheses. Genes up-regulated in LL are in light red while genes up-regulated in SS are in dark red. (File: Fig 5.tif).

Most of the differentially expressed genes in the LL *vs*. SS comparison were related to metabolic properties (Fig 5). Genes related to carbohydrate metabolism and energy generation were up regulated in LL, as expected in a fast-glycolytic muscle.

**Fig 6:**
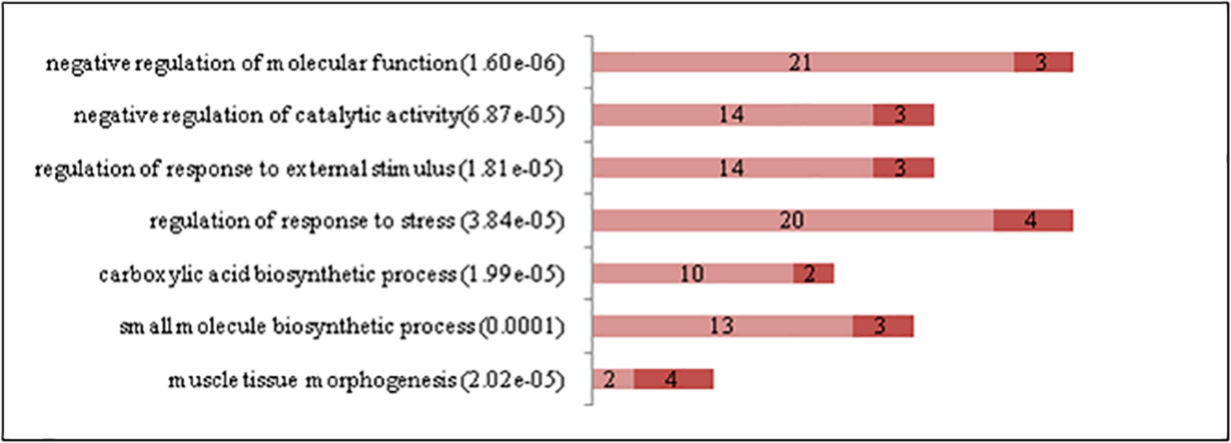
Gene Ontology biological process terms significantly enriched with differentially expressed genes between Semitendinosus (ST) and Gluteus medius (GM). The graphs show the number of differentially expressed genes in each functional category. P values are in parentheses. Genes up-regulated in ST are in light red while genes up-regulated in GM are in dark red. (File: Fig 6.tif).

With regard to the comparison ST *vs*. GM (S4 Table), several up regulated genes in ST are consistent with fast white muscles characteristics, including muscle contraction (*LMOD1*, [53] and *MYLK4* [54]), development (*PITX2* [55]), glucose homeostasis (*STXBP5L* [34]), signal transduction (*MPP6* [56]), protein phosphorylation and energy metabolism (*PRKAB2* [57]). Some of the genes up regulated in GM resemble the expression pattern of red muscles, such as *MYH7B*, *MYOZ2*, *RRAD* (the latter related to glycolysis inhibition and calcium channels regulation [58]) and *VEGFA* (vascular growth factor [59]. Most GO expression differences in ST *vs*. GM comparison were not associated with energy metabolism but to catalytic activity regulation and response to external stimuli (Fig 6).

With regard to meat quality, Ithurralde et al. (2017) reported that redness parameter a* was significantly higher (p < 0.05) in SS than in ST, LL, and GM, probably related to its oxidative metabolism [13]. SS also showed the highest yellowness parameter b*, followed by ST, which the authors relate to higher amounts of intramuscular fat and connective tissue with respect to other muscles. In this respect, it is interesting that *FABP3*, a gene that encodes a fatty acid transport enzyme, is one of the top 30 differentially expressed genes in SS (Table 1) and that lipid metabolic processes were up-regulated in ST (Fig 3).

Several genes that were more expressed in SS are related to cell adhesion and cytoskeleton organization (*PDLIM1*, *CCDC63*, *TPM3*, *MAST2* and *TACC2* [60, 29, 61, 62]) and extracellular matrix components (*FREM2* [63]), which in turn are associated with the high connective tissue content, high yellowness parameter and relatively high WBSF score (2.28 kg/cm^2^) found by Ithurralde et al. (2017) for this muscle [13]). Accordingly, in a comparison between 18 lamb muscles, [64] found that SS was one of the muscles with higher collagen content. The relatively high expression of genes involved in muscle contraction and development in SS (Figs 3–5) could be related to this.

Protein phosphorylation is negatively related to *post-mortem* tenderization [65]. Several genes involved in protein phosphorylation were found up regulated in SS, SM and ST, three muscles that showed high WBSF scores (2.28, 2.73 and 2.55 kg/cm^2^, respectively), in contrast with GM, a muscle with low WBSF (1.81 kg/cm^2^; [12]).

Higher glycolytic activities and lactic acid generation of fast or intermediate muscles tend to decrease meat pH values, being significantly lower in ST, LL, SM and GM than in SS (p < 0.05; [12]). This is in agreement with our findings that several carbohydrate and carboxylic acids (e.g. lactic acid) metabolic processes were up regulated in ST in comparison with SS (Fig 3), and with the high expression of *SLC16A3* in SM, a gene closely involved in lactate transmembrane transport [37].

### Validation of gene expression analysis

**Fig 7:**
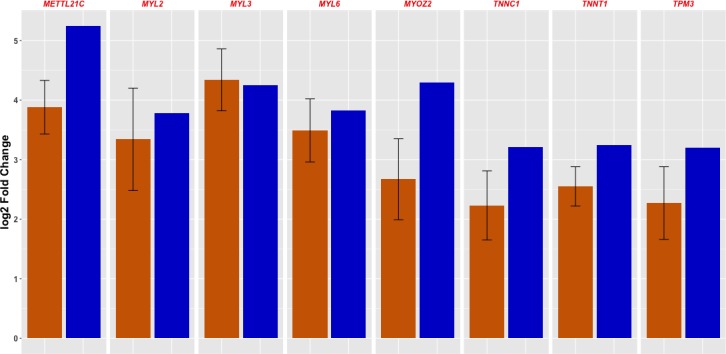
Fold changes of eight differentially expressed genes measured by RNA-Seq (blue) *vs*. qRT-PCR (dark orange). The eight genes showed higher expression in Supraspinatus (SS) *vs*. Semitendinosus (ST). Spearman correlation value = 0.84.

In order to validate the findings of the RNASeq analysis, the expression of eight genes, namely *MYOZ2*, *MYL2*, *MYL3*, *TPM3*, *MYL6*, *TNNT1*, *TNNC1* and *METTL21C*, was assessed using qRT-PCR. Fig 7 shows the fold differences in gene expression measured by both RNA-Seq and qRT-PCR, confirming that the eight genes showed similar patterns of mRNA abundance with both methods.

## Conclusions

Gene expression profiles obtained with RNA-Seq technology clearly separated muscles with different histochemical and metabolic properties. Pairwise comparisons between muscles show significant differences in terms of both major genes and relevant functions and processes closely related to meat quality parameters. Our results reveal new candidate genes associated with meat quality in sheep that deserve future research, and give a deeper insight into the genetic architecture of these complex traits.

## Supporting information

S1 Fig Schematic diagram showing the anatomical position of the studied muscles in a lamb carcass: SM: muscle Semimembranosus; GB: muscle Gluteobiceps; ST: muscle Semitendinosus; GM, muscle Gluteus medius; SS: muscle Supraspinatus. a) Transverse section of the hind limb showing muscles Rectus femoris (RF, cranial position), Gluteobiceps(GB, lateral position), Semitendinosus (ST, caudo-lateral position), Semimembranosus (SM caudo-medial position) and Adductor (AD, medial position). b) Transverse section of the loin showing muscles Longissimus lumborum (LL, dorsal position) and Psoas major (PM, ventral position).
(DOCX)

S1 Table Primers used for qPCR validation analysis. (DOCX)

S2 Table Summary of sequence read alignments to the reference genome. (XLSX)

S3 Table Top 30 most significant genes detected in Longissimus lumborum (LL) *vs*. Supraspinatus (SS) pairwise comparison. Positive log_2_ fold change (Log_2_FC) means higher expression in LM *vs*. SS.
(DOCX)

S4 Table Genes contributing to the Gene Ontology terms analyzed. Gene name: gene Ensembl ID.
(XLSX)
