# Supplementary material for: Comparison of transcriptomic landscapes of different lamb muscles using RNA-Seq
Source: PLoS One. 2018 Jul 24;13(7):e0200732. doi: 10.1371/journal.pone.0200732 (PMC6057623; doi:10.1371/journal.pone.0200732)
Supplement: S1 Fig — a) Transverse section of the hind limb showing muscles Rectus femoris (RF, cranial position), Gluteobiceps(GB, lateral position), Semitendinosus (ST, caudo-lateral position), Semimembranosus (SM caudo-medial position) and Adductor (AD, medial position). b) Transverse section of the loin showing muscles Longissimus lumborum (LL, dorsal position) and Psoas major (PM, ventral position). (DOCX) [file pone.0200732.s001.docx]

**S1 Figure:** Schematic diagram showing the anatomical position of the studied muscles in a lamb carcass: SM: muscle Semimembranosus; GB: muscle Gluteobiceps; ST: muscle Semitendinosus; GM, muscle Gluteus medius; SS: muscle Supraspinatus. **a)** Transverse section of the hind limb showing muscles Rectus femoris (RF, cranial position), Gluteobiceps(GB, lateral position), Semitendinosus (ST, caudo-lateral position), Semimembranosus (SM caudo-medial position) and Adductor (AD, medial position). **b)** Transverse section of the loin showing muscles Longissimus lumborum (LL, dorsal position) and Psoas major (PM, ventral position).
