# Supplementary material for: Comparison of transcriptomic landscapes of different lamb muscles using RNA-Seq
Source: PLoS One. 2018 Jul 24;13(7):e0200732. doi: 10.1371/journal.pone.0200732 (PMC6057623; doi:10.1371/journal.pone.0200732)
Supplement: S1 Table — (DOCX) [file pone.0200732.s002.docx]

**S1 Table: Primers used for qPCR validation analysis.**

| Gene | Primer sequence  (Fw/Rv) | Primer  size (bp) | Spanned exons | Amplicon  size (bp) |
| --- | --- | --- | --- | --- |
| Candidate genes* |  |  |  |  |
| *MYOZ2* | 5´ACCACGGTTACTGAGATGGG 3´  5´GGTGAAGCAGAGGAAAGAGC 3´ | 20  20 | 4  5 | 142 |
| *HOXD8* | 5´CCCCTTCTTCTACCAGGAGC 3´  5´ATATTGGCGAGGACCCAGAC 3´ | 20  20 | 1  2 | 97 |
| *MYL2* | 5´CTTTGGAAAACCTCTCCGCC 3´  5´AGACCATTCTCAACGCATTCA 3´ | 20  21 | 3  4 | 108 |
| *MYL3* | 5´TCTCCAAGAACAAGGACACCG 3´  5´ATCTTCTGTCAGCCTCTCACC 3´ | 21  21 | 4  5 | 140 |
| *TPM3* | 5´TCCGAATACTTGTCCAGCTCA 3´  5´CAGAAGCAGGCAGAAGAAAGA 3´ | 21  21 | 4  5 | 101 |
| *MYL6* | 5´GCCAAAACAGACTCCACCTC 3´  5´CAAAGTCATGGGAGCTGAGC 3´ | 20  20 | 3  4 | 84 |
| *MYBPH* | 5´CCTTGTCTTTCTGTCTGCCTT 3´  5´ATGTCTGGAACTGCAACGCTA 3´ | 21  21 | 4  5 | 104 |
| *TNNT1* | 5´GAAAGAGGAAGAGGAGCTGGT 3´  5´GGTTCTGAAGCGCTGTTGTTC 3´ | 21  21 | 3  4 | 85 |
| *TNNC1* | 5´AGGAGATGATTGACGAGGTGG 3´  5´CTTCATGCAGCGAACCATCAT 3´ | 21  21 | 3  4 | 86 |
| *METTL21C* | 5´TCGGAGATTGAGTTCCTCTGT 3´  5´CCAGCTACACTCAGGAGAACT3´ | 21  21 | 2  3 | 146 |
| Reference genes** |  |  |  |  |
| *ACTB* | 5´CCAACCGTGAGAAGATGACC 3´  5´CCAGAGGCGTACAGGGACAG 3´ | 20  20 | 2  3 | 97 |

Fw: forward primer; Rv: reverse primer; bp: base pairs.

*Designed for this study.

** [24]
