# Supplementary material for: Comparison of transcriptomic landscapes of different lamb muscles using RNA-Seq
Source: PLoS One. 2018 Jul 24;13(7):e0200732. doi: 10.1371/journal.pone.0200732 (PMC6057623; doi:10.1371/journal.pone.0200732)
Supplement: S3 Table — Positive log2 fold change (Log2FC) means higher expression in LM vs. SS. (DOCX) [file pone.0200732.s004.docx]

**S3 Table: Top 30 most significant genes detected in Longissimus lumborum (LL) *vs.* Supraspinatus (SS) pairwise comparison.** Positive log_2_ fold change (Log_2_FC) means higher expression in LM *vs.* SS.

| **Functional Characterization** | **Gene Name** | **Log_2_FC** | **P value** |
| --- | --- | --- | --- |
| Regulation of transcription; development and morphogenesis. | HOXD8 | 4.56 | 9.16E-40 |
| Carbohydrate catabolic process; protein phosphorylation. | CHI3L1 | 4.20 | 1.37E-29 |
|  | NTU | 4.79 | 4.38E-25 |
|  | NTU | 5.75 | 4.84E-24 |
| Transcription regulation; cell diferentiation; development. | SIM1 | 4.87 | 1.17E-23 |
| Adrenergic receptor signaling pathway; regulation of sodium channels; thermogenesis. | ADRB2 | -1.67 | 2.12E-23 |
| Muscle contraction. | SNTB1 | 2.27 | 1.15E-22 |
| Muscle fiber organization. | MYL6 | -3.41 | 9.48E-22 |
|  | NTU | 3.83 | 4.23E-21 |
| Cellular potassium ion homeostasis. | KCNMA1 | -1.46 | 4.60E-20 |
| Cytoskeleton protein; cell-cell adhesion. | PDLIM1 | -1.70 | 5.20E-20 |
| Muscle fiber development. | MYL2 | -2.79 | 6.89E-20 |
| Striated muscle tissue development; protein dephosphorylation and sumoylation. | EYA1 | -3.49 | 5.20E-19 |
| Carboxylic acid metabolic process. | GADL1 | 2.21 | 2.15E-18 |
|  | NTU | 4.72 | 3.99E-18 |
| Myofibril assembly; actin binding. | MYOZ2 | -2.29 | 3.06E-17 |
| Chromatin-mediated regulation of transcription. | PADI2 | 4.38 | 3.72E-17 |
| Development and morphogenesis; neuromuscular processes. | HOXC10 | 6.80 | 5.89E-17 |
|  | NTU | 3.06 | 1.15E-16 |
| Regulation of transcription and cell proliferation. | SMYD2 | 1.41 | 1.88E-15 |
| Chemical synaptic transmission; negative regulation of phosphatase activity. | DLG2 | -2.60 | 3.69E-15 |
| Cytoplasmic microtubules organization; intracellular transport. | CCDC88C | 1.54 | 5.22E-15 |
| RNA splicing; heart development. | RBM20 | 1.84 | 2.05E-14 |
| Regulation of ion transmembrane transport | KCNS3 | 2.59 | 2.10E-14 |
|  | NTU | 3.27 | 2.99E-14 |
|  | NTU | 2.96 | 6.71E-14 |
|  | NTU | 3.12 | 8.29E-14 |
| Regulation of synaptic transmission. | PLCL2 | 1.50 | 2.05E-13 |
| Cell adhesion. | CTNNAL1 | 1.17 | 2.21E-13 |
|  | NTU | 1.58 | 2.44E-13 |

NTU: Novel Transcript Unit.
